# Is oral microbiome of children able to maintain resistance and functional stability in response to short-term interference of ingesta?

**DOI:** 10.1007/s13238-020-00774-y

**Published:** 2020-08-17

**Authors:** Fangqiao Wei, Xiangyu Sun, Yufeng Gao, Haoyu Dou, Yang Liu, Lili Su, Haofei Luo, Ce Zhu, Qian Zhang, Peiyuan Tong, Wen Ren, Zhe Xun, Ruochun Guo, Yuanlin Guan, Shenghui Li, Yijun Qi, Junjie Qin, Feng Chen, Shuguo Zheng

**Affiliations:** 1 Department of Preventive Dentistry, Peking University School and Hospital of Stomatology, National Clinical Research Center for Oral Diseases, National Engineering Laboratory for Digital and Material Technology of Stomatology, Beijing Key Laboratory of Digital Stomatology, Beijing, 100081 China; 2 Central Laboratory, Peking University School and Hospital of Stomatology, National Clinical Research Center for Oral Diseases, National Engineering Laboratory for Digital and Material Technology of Stomatology, Beijing Key Laboratory of Digital Stomatology, Beijing, 100081 China; 3 Promegene Institute, Shenzhen, 518110 China; 4 Tsinghua-Peking Center for Life Sciences, Beijing, 100084 China

**Dear Editor,**

A series of studies had focused on the ecological stability of human microbiome (Lozupone et al., 2012; Faith et al., 2013; Moya and Ferrer, 2016). Despite the continuous perturbation and the highly personalized composition within the human microbiome (Human Microbiome Project, 2012), healthy adults stably maintain their microbial communities in terms of space and time (Faith et al., 2013; Moya and Ferrer, 2016; Oh et al., 2016). This stability is proved to be critical for the well-being of human body (Lozupone et al., 2012). On the contrary, major shifts in microbial community composition are often related to diseases (Lynch and Pedersen, 2016).

Of the many habitats throughout the human body, the bacterial community of the oral cavity is highly complex, constituting the second most diverse microbiome in the human body (Human Microbiome Project, 2012). These bacteria have a wide variety of functions, and many of them are important in maintaining oral health (Gao et al., 2018). Moreover, it is proved that some of the oral bacteria could colonize and persist to live in the gut, suggesting that oral bacteria might contribute to the emergence of chronic inflammatory diseases in gut (Lira-Junior and Bostrom, 2018). Oral microbiome dysbiosis is associated with oral diseases such as caries and periodontal diseases. Furthermore, a number of systemic diseases is associated with the dysbiosis of oral microbiome, including diabetes, rheumatoid arthritis and Alzheimer’s disease (Gao et al., 2018). Because oral cavity is the gateway to the human body for both food and air intake with frequent disturbances undergone, it is important to figure out whether oral microbiome can maintain stability in daily life within a short period.

Considering the rapid and evenly distributed effects of beverages and the same standard schedule in kindergarten, we took three kinds of popular beverages as representatives of ingesta (orange juice [Group J], sugar-free tea [Group T], sugar-free liquid yoghurt [Group Y], representing the effects of sugar, tea polyphenols, and exogenous bacteria; and water [Group W] as control) to design a study to investigate the change of the oral microbial communities of children and their functional variations, using *16S rRNA* gene, metagenomic sequencing and the Biolog technology, to further explore the characteristics of the human oral microbiome in maintaining stability in response to beverage intake (Fig. 1A). It was hypothesized that the species composition of oral microbiome could resist the interference of short-term beverage intake with a quick recovery to maintain functional stability.

**Figure 1:**
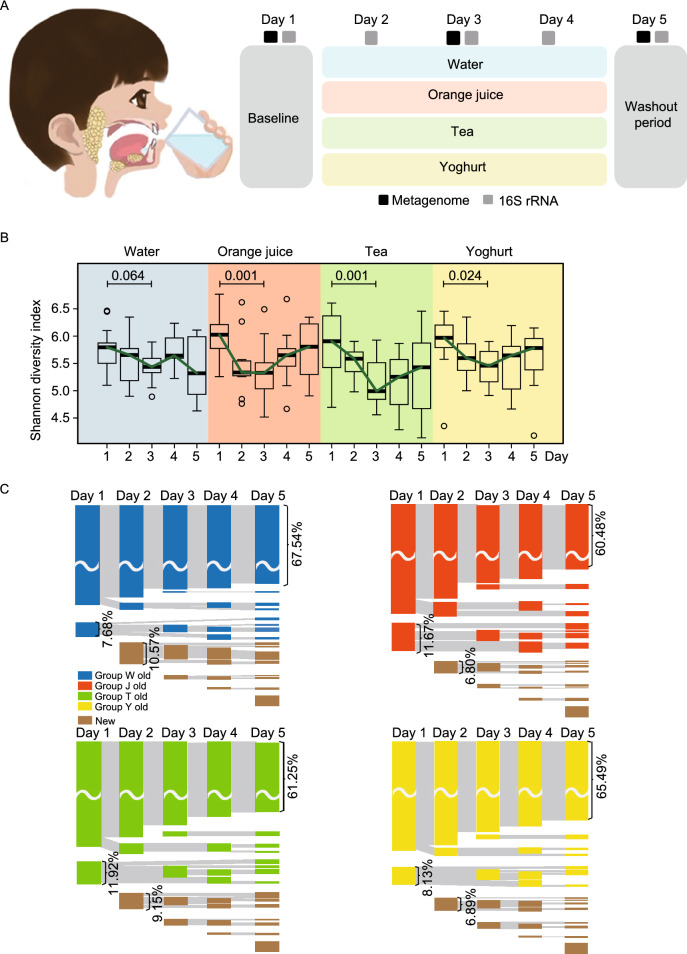

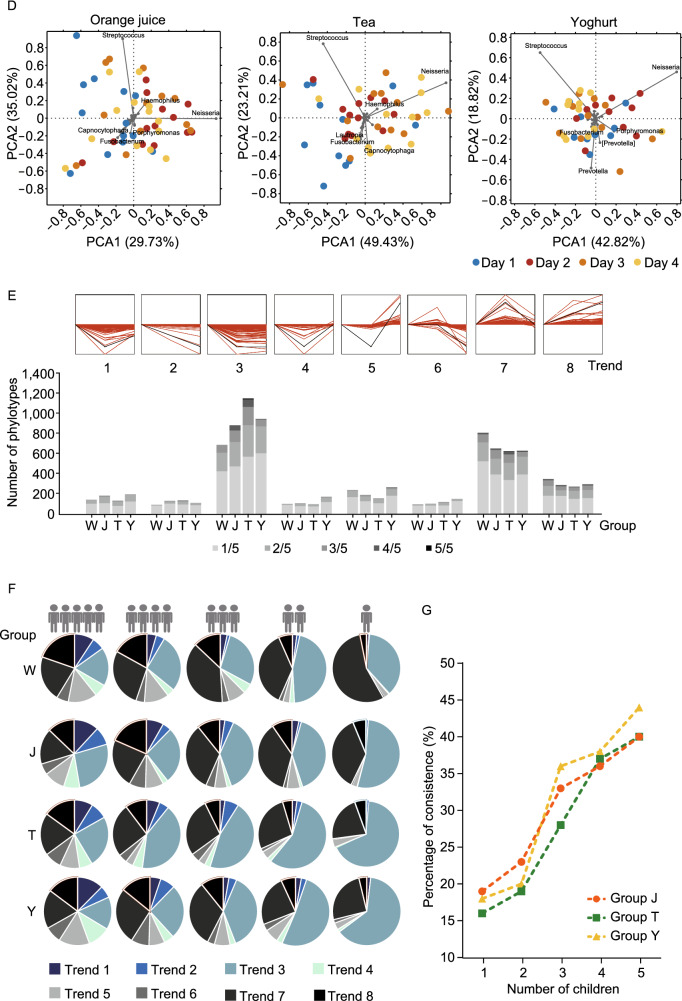
**The variations of carriage of microbial taxa during short-term intake of ingesta.** (A) The short-term longitudinal study design and sampling orders of beverages. (B) Diversity comparisons in terms of the Shannon indices by *16S rRNA* sequencing of the oral flora from the 4 groups over the 5 days. Boxplots exhibit the median and IQR, with whiskers extending to the last data points. (C) Sankey diagram showing the dynamic processes of oral bacterial genera in the 4 groups. The blue, red, green and yellow bars represent the genera from different groups which present originally but disappeared later. The brown bar represents newly detected genera. The same genera on different days are linked with grey lines. The width of links is proportional to the number of genera. (D) PCA analysis of the baseline and the interferent period of Groups J, T and Y. The colored rings stand for samples, while the black spots show the main genera contributed. (E) The 8 categories of trends and the distribution of number of shared individuals in each trend among 60 samples. “1/5” represents that among the five individuals, one of them fell in this trend; “2/5”, two of the five individuals had this trend; so as “3/5”, “4/5” and “5/5”. (F) Pie graphs of the distribution of trends based on the number of shared individuals. (G) The proportion of trends consistent with the control group in the other three groups with interference (Groups J, T and Y). All the phylotypes were grouped by the number of shared individuals

We first focused on the resistance of oral microbiome to beverage interference. Initially, we investigated the changes of oral microbiota in response to several factors using permutational multivariate analysis of variance (PERMANOVA) (Fig. S1A). According to the analysis, beverage intake was closely associated with the constitution of salivary microbial communities second to individual specificity. We also carried out a single nucleotide polymorphism (SNP)-based phylogenetic analysis to assess differences among the samples at the strain level. The sequences representing the top-9 abundant phylotypes from the same individual shared the most SNPs, so most samples from the same person were adjacent to one another in the phylogenetic analysis (Fig. S1B), indicating that an individual’s microbiome profile could be relatively stable over time though short-term beverage consumption altered the salivary microbial communities. This stable state was found in a number of sites in the healthy population (Human Microbiome Project, 2012; Lloyd-Price et al., 2017).

In the diversity analysis, Shannon indices of all groups with interference showed a significantly decrease after beverage consumption, while the control group exhibited a narrower range (Fig. 1B). Interestingly, the Shannon indices reached their lowest levels on day 3 in Groups J, T and Y, and showed an increase on day 4, suggesting a recovery of taxon number during the consumption. To figure out the constitution of the recovered taxon, we studied the dynamic process by Sankey diagram (Fig. 1C). As the diagram showed, the numbers of disappeared genera declined from day 2 to day 4, and all the groups with interference had more genera disappeared when compared with Group W. A certain number of genera that disappeared during the beverage consumption exhibited a reappearance on day 3 and 5. The newly-detected genera also had a decline trend from day 2 to day 4. A similar change-recover pattern in the microbiome structure was shown by Principal component analysis (PCA) based on the data of day 1, 2, 3, and 4 (Fig. 1D). The spots of day 4 was closer to the baseline than those of day 2 or day 3 in Groups J and Y. Recently, a theory of multi-stable community was put forward to explain the maintenance and changes of the stable state (Gonze et al., 2017), suggesting that the microbial system would keep its original state until reaching a tipping point of microbiome. In this context, we speculated that oral microbiome’s “tipping point” was beyond the reach of short-term intakes of ingesta. Based on previous reports, the resistance to diet change in gut microbiome was found (Lang et al., 2018), whereas the skin microbiome could maintain a high level of stability (Oh et al., 2016). It was also reported that higher diversity could provide greater functional resilience to perturbations (Lang et al., 2018). All these evidence suggest that saliva, as a body fluid which contained microbes from all sites of oral cavity and formed a comparatively diverse microbiome (Simon-Soro et al., 2013), might be an advantageous factor for the rapid recovery.

To investigate the specific variation at the phylotype level, we carried out a short time-series expression miner (STEM) analysis to classify the phylotypes into 8 trends according to their changing patterns based on the metagenomic data. Of the 5 individuals in each group, each phylotype has a series of all 8 trends. As shown in Fig. 1E, most of the phylotypes had no preponderant trend, and trend 3, 7, and 8 were the top-three trends in all the groups. We ranked all the phylotypes by the number of shared individuals and draw a pie graph to show the distribution of phylotypes of all the rankings, which indicated that for phylotypes shared by all the five children (i.e., “common phylotypes”), the groups with interference had a similar and evenly distributed characteristic with the control group (Fig. 1F). As the number of shared individuals declined, the groups with interference exhibited more distinct differences with Group W in distributions, especially for those phylotypes found only in one individual. Comparing to Group W, more unrecovered trends and fewer recovered trends were found in phylotypes shared only by one or two children. After that, we compared the trends of each phylotype with the control group, and calculated the proportion of trends consistent with the control (Fig. 1G). As a result, we found that the proportion of consistent trends grew from 15%–20% to 40%–45% when the number of shared individuals was rising, suggesting that the common phylotypes were less disturbed and contributed less to the change of oral microbiota than other phylotypes. Since salivary microbiome comprised microbes from almost all sites around the oral cavity, among which biofilms constituted an important part (including but not limited to dental plaque and tongue). These biofilms consisted of relatively constant components of bacteria (Bowen et al., 2018), which greatly contributed to the composition of common phylotypes. As the fact that these common phylotypes accounted for 99.13%–99.51% of the oral microbiome, along with their imperturbable nature against beverage intake, the evenly distributed trends of common phylotypes ought to play a critical role in the resistance of oral microbiota against short-term beverage intake.

Then we investigated the changes in each group with interference. In Group J, oral microbiome had a greater variation and a faster recovery than other beverages (Fig. S2A and S2B). As for Group T, the resistance to interferent was not that obvious compared with Group J and Y in the result of PCA (Fig. 1D), and Group T had more phylotypes belonged to trend 3 than other groups (Fig. 1E), containing a much higher proportion of phylotypes with the abundance under 0.0001 (Fig. S2C). These results suggested that the resistance against tea may be less than orange juice and yoghurt. Although the impact of exogenous bacteria (especially for probiotics) was reported by a number of previous studies on human microbiome, yoghurt had little impact on salivary microbiome according to our findings. The heatmaps of phylotypes with a trend shared by no fewer than three individuals among the 5 children were shown in Fig. S3.

Following these investigations on species composition, our second focus was the functional stability of oral microbiome to beverage interference. Unlike the variations found at the genus level (Fig. S4), the composition at the pathway level was less affected by the intake of ingesta (Fig. 2A). Our findings suggested that all the children shared a similar and stable pathway pattern.

**Figure 2:**
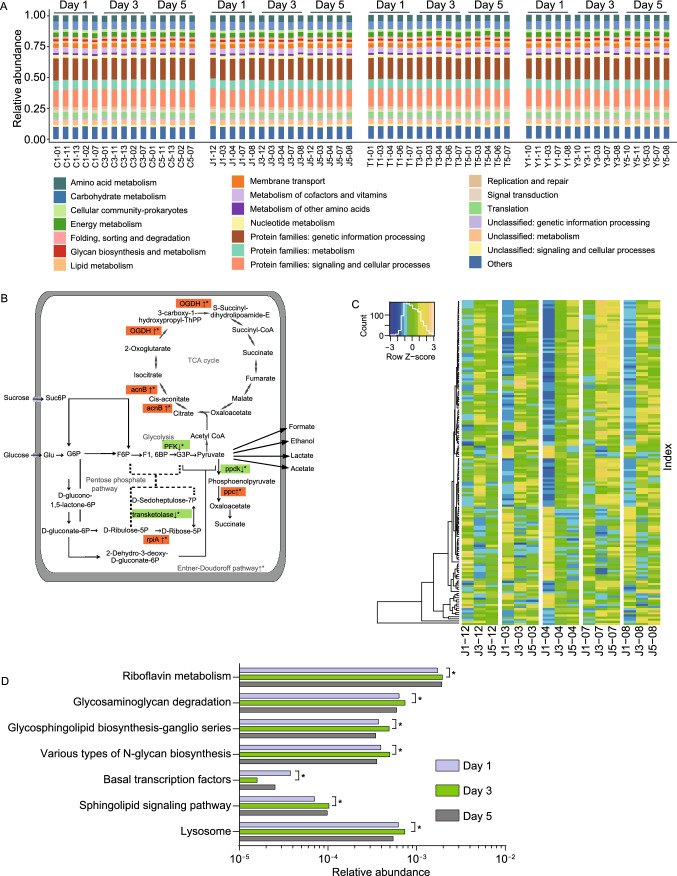

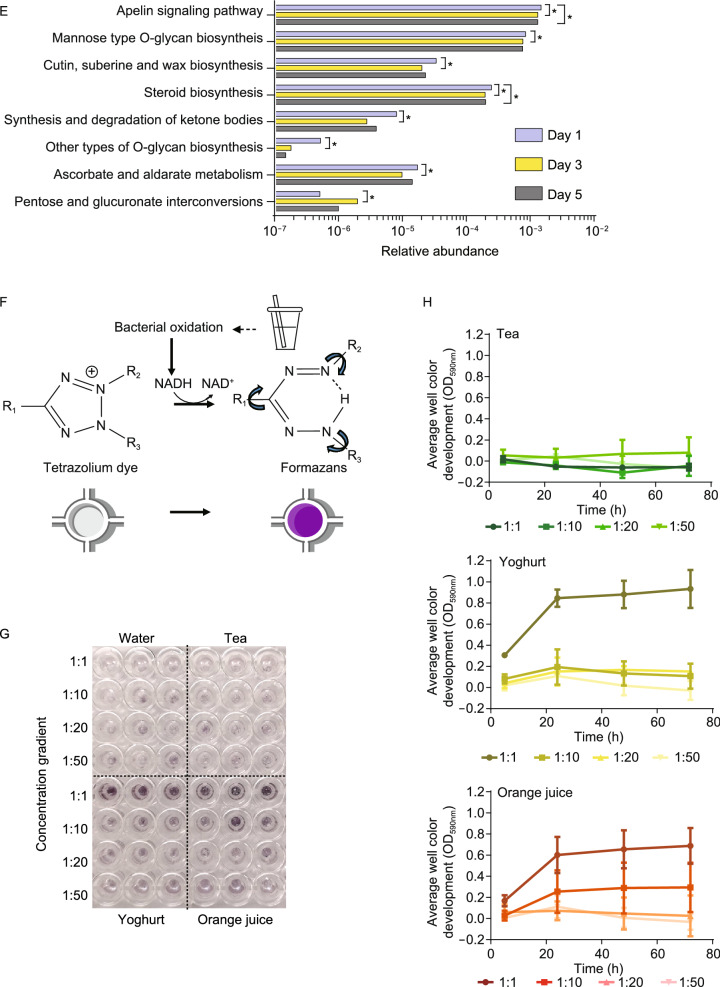
**The functional redundancy of oral microbiome facing intakes of ingesta and the saliva samples incubated with beverages using the Biolog technology.** (A) The metabolic pathways remain stable during short-term beverage intake. Vertical bars represent microbiome samples in day 1, day 3, and day 5 with metagenomic data; bars indicate relative abundances colored by metabolic pathways at level 2 (the top 20 abundant categories were shown). (B) The changes of some significantly changed KOs in central carbohydrate metabolism in group J. Asterisks indicate significant differences (*P* < 0.05). OGDH, 2-oxoglutarate dehydrogenase E1; acnB, aconitate hydratase 2; PFK, 6-phosphofructokinase; ppdk, pyruvate orthophosphate dikinase; ppc, phosphoenolpyruvate carboxylase; rpiA, ribose 5-phosphate isomerase A; Glu, glucose; Suc6P,; G6P, D-Glucose-6P; F6P, D-Fructose-6P; F1,6BP, D-Fructose-1,6P2; G3P, Glyceraldehyde-3P. (C) Unsupervised hierarchical clustering of significantly changed KOs between days 1 and 3 in Group J (*P* < 0.05). The KOs were listed in Table S2 with the same order. (D and E) Significantly changed pathways at level 3 in Group T and Y (*P* < 0.05). (F) Color reaction principle of this technology. In the redox reaction process, a tetrazolium dye was reduced by electrons from NADH produced by bacteria, resulting in irreversible formation of formazan product with a purple color. (G) Full view of the Biolog MT2 plate with mixed saliva samples incubated with beverages for 72 h. (H) The changes of AWCD values over time in the 3 groups (mean ± SD, dilution ratios were presented in form of color gradation)

To investigate whether the beverages affect oral microbial metabolic pathways, Kyoto Encyclopedia of Genes and Genomes (KEGG) database were used to identify differential KEGG orthologue groups (KOs). According to the analytical test report, orange juice contains a large amount of fructose and glucose (Table S1). Due to the high concentration of sugar, we studied the genes involved in carbohydrate metabolism in Group J. However, only 7 significantly changed KOs in the central carbohydrate metabolism were found, they were shown in Fig. 2B. In Fig. 2C, a recovery to the baseline level in the washout period was found in most of the significantly change KOs. As for Group T, among 5,784 KOs, only 4 KOs significantly changed, namely K00558, K02479, K07323, and K07481. Meanwhile, all the 7 significantly changed KEGG level-3 functional pathways between day 1 and 3 showed no statistical differences between day 1 and 5 (Fig. 2D). In Group Y, no significantly changed KOs were found. Most of the KEGG level-3 functional pathways that significantly changed between day 1 and 3 showed no statistical differences between day 1 and 5 (Fig. 2E). The functional redundancy could be very important because species were interchangeable in terms of functions (Human Microbiome Project, 2012; Lozupone et al., 2012; Moya and Ferrer, 2016), that is to say, phylotypes could have similar functions in the community while significant changes in microbiota might cause little variation to the functional outcome (Moya and Ferrer, 2016). This phenomenon was previously found in gut, and now we confirmed its validity for the oral microbiome.

Besides changes in species composition and functional pathways, the amounts of bacteria in saliva were also a point of focus worth studying as it could influence the stability of oral microbiome as well. To that end, an *in-vitro* experiment was designed to measure the amounts of bacteria in saliva samples incubated with the 3 beverages and water with a concentration gradient by tracing the changes of optical density (OD) values using the Biolog technology in 4 designate time points from 5 h to 72 h (Fig. 2F and 2G). The average well color development (AWCD) value of each group was calculated using observed value minus that of Group W at the same time point and with the same dilution ratio before statistical analysis commenced (Fig. 2H). In Group T, no significant proliferation was found among AWCD of the 4 timepoints regardless of beverage concentration (*P* > 0.05). As for the other two groups with interference (orange juice and yoghurt), bacteria commenced proliferating at the beginning and plateaued after 48 h only if the beverage was added to saliva without dilution (*P <* 0.05); however, when the dilution ratio became higher than 10, no significant proliferation was observed. Based on previous studies’ findings that the concentration of sucrose or citric acid would be diluted for 1,000–10,000 times after 15 min in saliva (Lagerlöf and Dawes, 1985; Bashir et al., 1995), and in combination with our findings of resistance and functional stability of oral microbiome against beverage interference, it was reasonable that lower concentration of beverages had less impact on oral microbiome, but this state might vary if beverages are taken more frequently. This indicated the significance of keeping lower concentration of beverages on maintaining the stable state of oral microbiome, directing a future research orientation to explore the changes of oral microbiome confronting long-term or persistent interference of ingesta, which might result in substantial influence on the characteristics of oral microbiome.

In summary, we found that salivary microbiota had a strong resistance and functional redundancy during the intake of ingesta, and the common phylotypes played an important role in maintaining the stability. Oral microbiome exhibited recoverable changes after short-term orange juice intake, while minor changes were found after drinking green tea and sugar-free liquid yoghurt. These findings would be contributory to the current knowledge of the oral microbiome and its prompt reaction to typical stimuli, and potentially be helpful to inform further research on the oral and salivary microbiome and the possibility of using saliva samples in health surveillance and clinical settings.

## Electronic supplementary material

Below is the link to the electronic supplementary material.

Supplementary material 1 (PDF 772 kb)
