# Supplementary material for: Is oral microbiome of children able to maintain resistance and functional stability in response to short-term interference of ingesta?
Source: Protein Cell. 2020 Aug 17;12(6):502–10. doi: 10.1007/s13238-020-00774-y (PMC8160059; doi:10.1007/s13238-020-00774-y)
Supplement: Supplementary file 1 — Supplementary material 1 (PDF 772 kb) [file 13238_2020_774_MOESM1_ESM.pdf]

Materials and Methods

Study participants

More than 190 children (aged 3~5 years) were initially recruited from one kindergarten in Chaoyang District, Beijing. Inclusion and exclusion criteria were based on Human Microbiome Project – Core Microbiome Sampling Protocol A (http://www.hmpdacc.org/doc/HMP_Clinical_Protocol.pdf) with the following modifications: (i) no current oral disease or history of dental treatment; (ii) no antibiotic use within the past month; (iii) no upper respiratory infections within the past 2 weeks; and (iv) no systemic diseases. In total, 60 children were included, and 49 of them (Supplementary Table 2) finished the trial (23 boys and 26 girls; mean age, 3.55 ± 0.50 years), while 11 were excluded because of absence, insufficient saliva, or bloody saliva. All of these children had attended the kindergarten and underwent the same standard schedule (including meals and exercise) for at least 7 months, which minimized inter-personal variation.

Sample collection

The details of the selected ingesta (beverages) are listed in Table S1. Children were grouped into Groups J, T, Y, and W in similar numbers, gender ratios, and age composition (Table S3). Sampling lasted for five consecutive days in April 2014 (Monday to Friday), with the first and last day set as the baseline and washout periods, respectively. We selected weekdays to reduce the impact of the specific dietary habits of different families during weekends. Beverage intake occurred from day 2 to day 4,

during which ~80 mL of the corresponding beverage was given to each child at four time points (09:30, 10:00, 11:00, and 14:00). They were instructed to only drink water during the 5 days except the 3 beverages. Whole stimulated saliva (~2 mL) was generated through passive tooth-brushing by trained dentists from Peking University School and Hospital of Stomatology at 14:30 each day (Fig. 1). The samples were collected in sterile, ice-chilled tubes and placed in liquid nitrogen for temporary storage. One milliliter of saliva was then aspirated and centrifuged at 10,000 × *g* for 10 min at 4 ℃. The pellets were stored at –80 ℃ until DNA extraction.

DNA extraction and 16S *rRNA* V3–V4 region sequencing

DNA was extracted using the TIANamp Bacteria DNA Kit DP302 (Tiangen, Beijing, China) according to the manufacturer's protocol. DNA purity was evaluated based on the A260/A280 ratio using a NanoDrop 8000 Spectrophotometer (Thermo Fisher Scientific, Wilmington, NC, USA). DNA integrity was verified by agarose gel electrophoresis. A negative control containing only buffer was included during DNA extraction and quantification. DNA was stored at –80 ℃ in Tris-EDTA buffer before use. To enable the amplification of the V3–V4 region of the *16S rRNA* gene and the addition of barcode sequences, we designed unique fusion primers based on the universal primer set, 338F (5′-GTACTCCTACGGGAGGCAGCA-3′) and 806R (5′-GTGGACTACHVGGGTWTCTAAT-3′), along with barcode sequences. The PCR mixtures contained 1 μL of template DNA (30 ng), 2 μL of each forward and reverse primer (10 μM), 4 μL of deoxynucleotides (2.5 mM), 5 μL of 10× Pyrobest buffer, 0.3 μL of Pyrobest DNA polymerase (2.5 U/μL, TaKaRa Code: DR005A), and

35.7 μL of ddH_2O in a 50 μL reaction volume. Thermal cycling consisted of an initial denaturation step at 95 ℃ for 5 min, followed by 25 cycles of denaturation at 94 ℃ for 30 s, annealing at 57 ℃ for 30 s, and extension at 72 ℃ for 40 s, with a final extension step at 72 ℃ for 4 min. PCR amplicons were subsequently purified using the PCR Product Rapid Recovery and Purification Kit (ZhiAng Biotechnology, Changchun, China). Quality was assessed by agarose gel electrophoresis after the first two steps. An amplicon library for high-throughput sequencing on the Illumina MiSeq platform was constructed and subsequently quantified (KAPA Library Quantification Kit KK4824) according to the manufacturer's instructions.

qPCR for sample selection and metagenome sequencing

The human DNA in samples collected on days 1, 3, and 5 was quantified by real-time quantitative polymerase chain reaction (qPCR) on an ABI 7500 Real-time PCR System (Applied Biosystems, Foster City, CA, USA) prior to sequencing, using human β-actin primers (5′-CGGGAAATCGTGCGTGAC-3′ [sense] and 5′-CAGGCAGCTCGTAGCTCTT-3′ [anti-sense]) as control. Sixty samples from 20 individuals (5 children from each group) with lower levels of overall human DNA content were selected for whole-genome sequencing (WGS). The DNA library was constructed following the manufacturer’s instructions (Illumina). Paired-end metagenomic sequencing was performed on the Illumina platform (insert size, 400 bp; read length, 125 bp). Low-quality and host reads were removed, and the remaining high-quality reads were used for further analyses.

Profiling of 16S rRNA gene sequencing data

Using the Quantitative Insights into Microbial Ecology pipeline (Caporaso et al., 2010), the raw sequences were processed to concatenate reads into tags according to their overlaps, after which reads belonging to each sample were separated with barcodes and low-quality reads were removed. The processed tags were clustered, and chimaeras were removed prior to analysis. Operational taxonomic units (OTUs) were defined at 97% sequence similarity to taxa by matching to the Greengenes database (DeSantis et al., 2006).

Taxonomic assignment of metagenomic data

A total of 32,605 sequenced bacterial genomes available in GenBank as of June 2015 were downloaded from the NCBI database (http://www.ncbi.nlm.nih.gov/). Clade-specific marker genes were identified, and all genomes were clustered into phylotype groups according to gene similarities using the procedure reported by a previous study (Segata et al., 2012).

Mantel test

A Mantel test was carried out to assess the correlation between 2 matrices, the *16S rRNA* OTU profile and the metagenome abundance profile. The test was processed by R software (3.3.1, vegan package; permutations = 999, *P* value = 0.001).

KEGG annotation

The catalogue of non-redundant genes was translated to putative amino-acid sequences and aligned with the proteins/domains in the KEGG database using usearch ublast algorithm 9 (Version v9.2.64, *e*-value < 1e-5, query coverage > 0.70). Each protein was assigned to one KO based on the highest-scoring annotated hit(s) containing at least one segment pair scoring >60 bits. The Wilcoxon rank-sum test (P value < 0.05) was used to identify significantly changed KOs and KEGG level-3 functional pathways.

PERMANOVA analysis

Based on the profiles of *16S rRNA* genes and metagenomic phylotypes, PERMANOVA assessed by 999 permutations for Bray distance was used to estimate the variation effects (R package vegan). In the analysis to compare the effects of beverages, the profile of metagenomic phylotypes was split by beverage, and the PERMANOVA interpretation of randomly-ordered time factors (999 permutations, Bray distance) was calculated for 1000 iterations. The PERMANOVA interpretation value of the time factor in its actual chronological order for each beverage was then compared with the distribution of the randomly-ordered time interpretation values.

SNP-based phylogenetic analysis

We selected the 9 most abundant phylotypes, each accounting for >2% of all known microbes generated from the metagenomic data, and then aligned the reads from all 60 samples to the genomes of strains representing the selected phylotype groups using the Burrows-Wheeler Aligner 0.7.15 (Li and Durbin, 2009). SNPs were called using Samtools 0.1.19.(Li et al., 2009) SNPhylo software (Lee et al., 2014) was used to

remove low-quality data and generate a phylogenetic tree with a minor allele frequency of 0.05.

Analysis of alterations in genus counts as indicated by 16S rRNA

Samples with complete time sequences were selected to determine the retained genus counts from day 1 to day 5. Divided according to the four beverages, the mean values of genus counts were calculated and visualized in the form of a Sankey diagram using R software (3.3.1, networkD3 package).

PCA

R software (3.3.1, ade4 package) was used to perform PCA on *16S rRNA* genus profiles for the different beverages.

STEM and correlation analysis

The relative abundance data of all the phylotypes were used to cluster time-series dynamic patterns by STEM (Bar-Joseph, 2006). The maximum number of clusters was 8, and the maximum unit change between time points was set to 2. Significance was calculated using the Wilcoxon rank-sum test (*P* value <0.05) between days 1 and 3, and between days 1 and 5.

Biolog assays and data analysis

Whole stimulated saliva (~1mL) of another 3 children was generated through passive tooth-brushing by trained dentists at 9:00 (Table S4). The samples were collected in sterile, ice-chilled tubes and placed in ice for temporary storage. One milliliter of saliva was then aspirated from each sample and all of them were mixed together. The

mixed saliva sample was centrifuged at 500 rpm for 30s to remove food debris, with the supernatants resuspended in 9mL PBS (0.01M, pH=7.2-7.4) and vortexed thoroughly for 60s. Beverage solutions with concentration gradients of 100%, 10%, 5%, and 2% were obtained by diluting the original beverage with PBS (0.01M, pH=7.2-7.4). After measuring ODs at baseline, 99μL saliva and 1μL beverage were inoculated into each well on 96-well Biolog MT2 plates (Biolog Inc.) which contained redox dye and a buffered nutrient medium optimized for a wide variety of bacteria. For the sake of repeatability, the experiment for each concentration was performed for three times. The plates were incubated in a 5% CO_2 incubator at 37 C ° for up to 4 days. The OD value at wavelength of 590 nm (OD590) in each well was recorded after 5h, 24h, 48h and 72h using a Biolog micro-station and software kit (Biolog OmniLog version 4.1).

All Biolog assay data were analyzed using SPSS 20.0 software (IBM, Armonk, NY). Differences in OD values among the 4 time points were evaluated using one-way ANOVA test. The threshold for statistical significance was set at $P<0.05$.

REFERENCES

Bar-Joseph, J.E.Z. (2006). STEM: a tool for the analysis of short time series gene expression data. BMC bioinformatics 7, 191.

Caporaso, J.G., Kuczynski, J., Stombaugh, J., Bittinger, K., Bushman, F.D., Costello, E.K., Fierer, N., Pena, A.G., Goodrich, J.K., Gordon, J.I., *et al.* (2010). QIIME allows analysis of high-throughput community sequencing data. Nat Methods 7, 335-336.

DeSantis, T.Z., Hugenholtz, P., Larsen, N., Rojas, M., Brodie, E.L., Keller, K., Huber, T., Dalevi, D., Hu, P., and Andersen, G.L. (2006). Greengenes, a chimera-checked 16S rRNA gene database and workbench compatible with ARB. Appl Environ Microbiol 72, 5069-5072.

Faith, J.J., Guruge, J.L., Charbonneau, M., Subramanian, S., Seedorf, H., Goodman, A.L., Clemente, J.C., Knight, R., Heath, A.C., Leibel, R.L., *et al.* (2013). The long-term stability of the human gut microbiota. Science 341, 1237439.

Lee, T.H., Guo, H., Wang, X., Kim, C., and Paterson, A.H. (2014). SNPhylo: a pipeline to construct a phylogenetic tree from huge SNP data. BMC Genomics 15, 162-167.

Li, H., and Durbin, R. (2009). Fast and accurate short read alignment with Burrows-Wheeler transform. Bioinformatics 25, 1754-1760.

Li, H., Handsaker, B., Wysoker, A., Fennell, T., Ruan, J., Homer, N., Marth, G., Abecasis, G., and Durbin, R. (2009). The Sequence Alignment/Map format and SAMtools. Bioinformatics 25, 2078-2079.

Segata, N., Waldron, L., Ballarini, A., Narasimhan, V., Jousson, O., and Huttenhower, C. (2012). Metagenomic microbial community profiling using unique clade-specific marker genes. Nat Methods 9, 811-814.

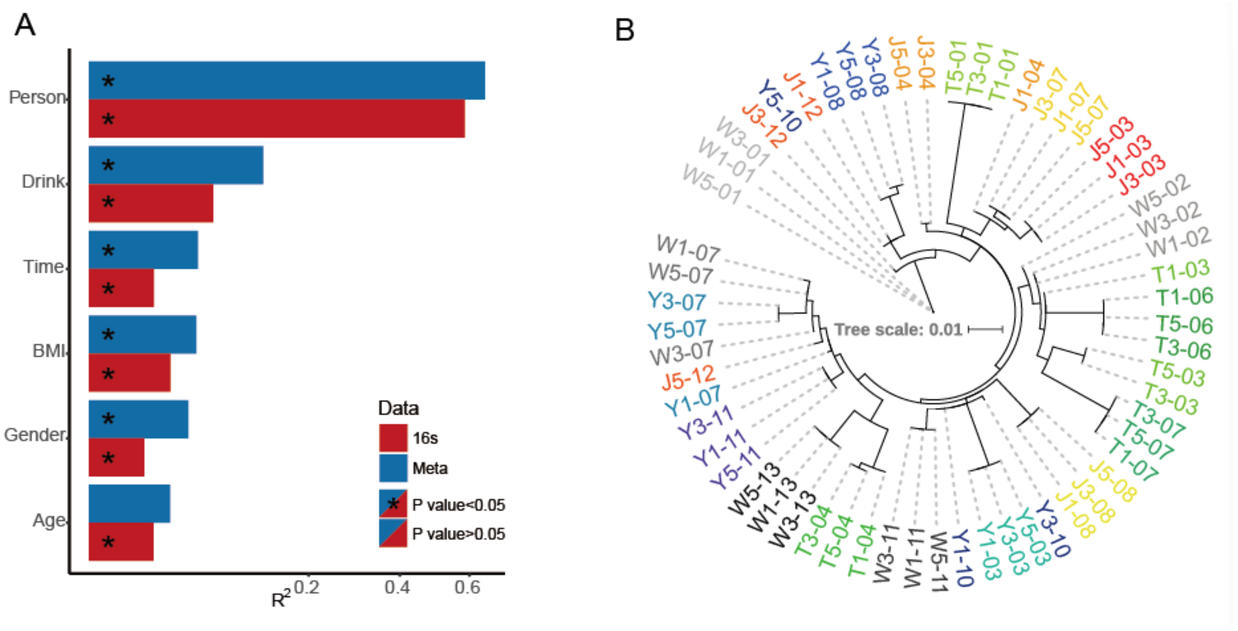

Figure S1. (A) PERMANOVA analysis for the influence of individual, beverage intake, gender, BMI, and age on oral microbiota. **(B)** Phylogenetic tree based on SNPs in sequences that represent the 9 most abundant phylotypes (relative abundance > 2%) based on WGS data. The 20 studied individuals at the three time points are marked by different colors.

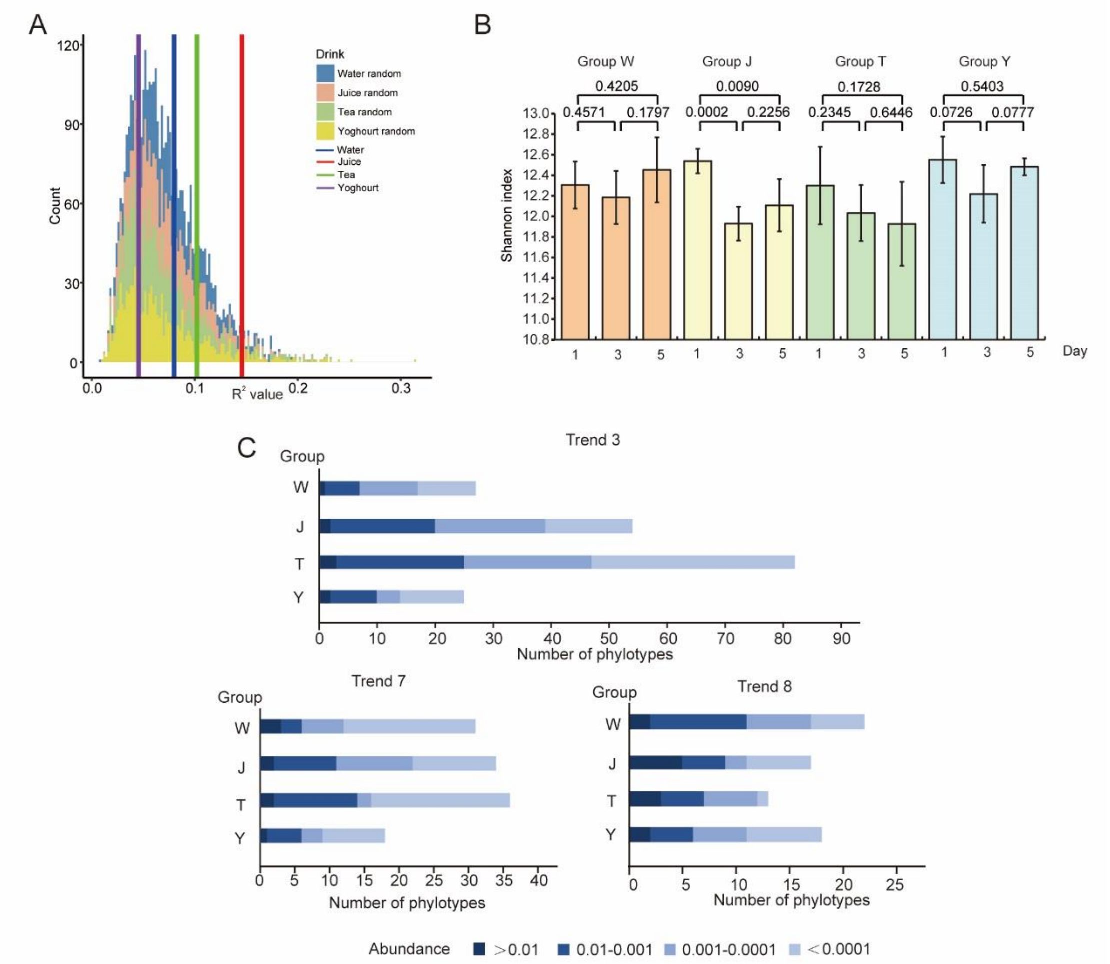

Figure S2. (A) PERMANOVA of the four beverages. The bars represent PERMANOVA interpretation (999 permutations, Bray distance) of the randomly ordered time factor for 1000 iterations, and the line represents the distribution of interpretation values for the data in chronological order. **(B)** Comparisons of diversity in terms of Shannon indices by metagenomic sequencing of the oral flora from the 4 groups over the 3 days. Boxplots show the median and IQR. **(C)** The distribution of phylotypes based on abundance in trend 3, 7, and 8 in all the four groups.

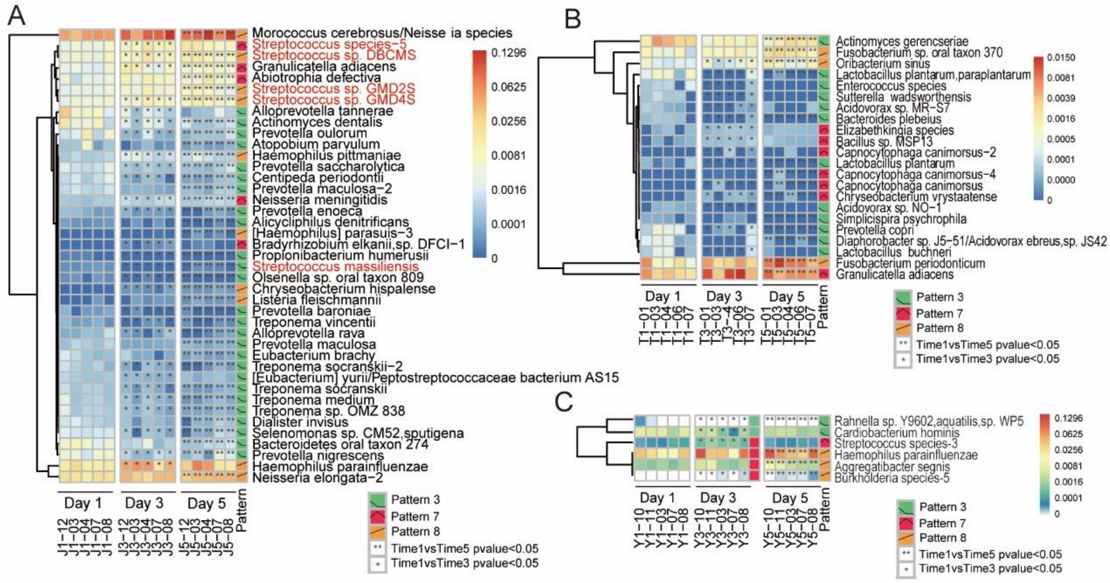

Figure S3. Species changes in the groups with interference. Heatmaps of significantly changed phylotypes with a trend shared by no fewer than three individuals among all the five children in Group J **(A)**, Group T **(B)**, and Goup Y **(C)**. Asterisks indicate significant differences (P < 0.05).

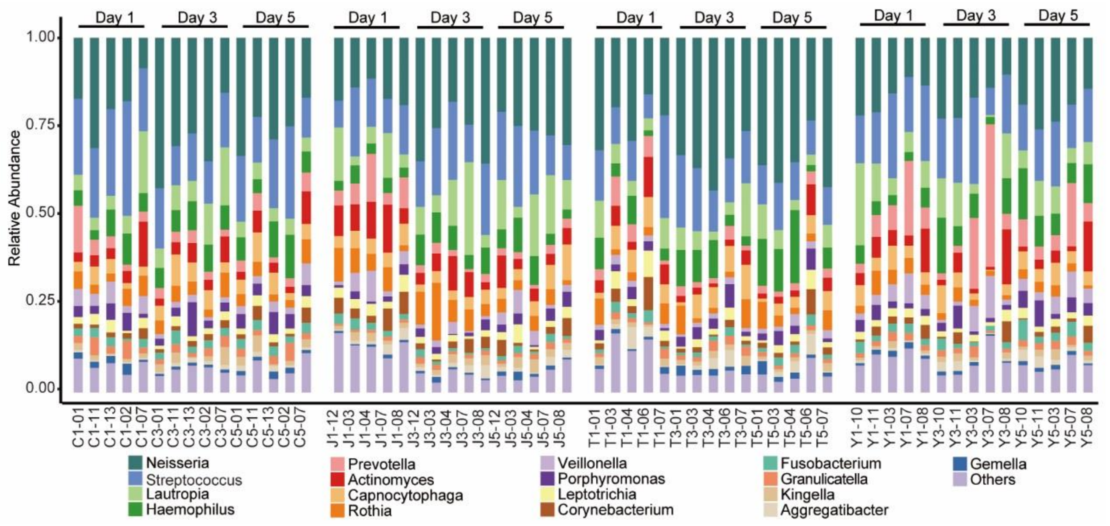

Figure S4. The variations of microbial taxa during short-term interference of ingesta. Vertical bars represent microbiome samples in day 1, day 3, and day 5 with metagenomic data; bars indicate relative abundances colored by microbial genera with an abundance higher than 0.01.

Table S1. Information of ingesta (beverages)

Group	Company	Brand	sugar	Tea polyphenols	Probiotics	Carbohydrate	Protein	Fat	Others
Orange juice drink	Lotte	Lilicheng	High fructose corn syrup，mandarin pulp; concentrated juice	—	—	12.7g/100ml (glucose: 33.8g/kg; fructose: 55.3g/kg; sucrose＜2g/kg)	0g/100ml	0g/100ml	Water, citric acid, sodium citrate, sodium carboxymethyl cellulose, vitamin C, food flavor
Tea drink	Nongfuspring	Dongfangshuye (green tea)	—	Yes	—	0g/100ml	0g/100ml	0g/100ml	Food flavor, food additives (vitamin C, sodium bicarbonate);
Yoghurt	Sanyuan	Yijunduo	—	—	*Streptococcus thermophilus*<br>*Lactobacillus bulgaricus*<br>*Lactobacillus acidophilus*<br>*Bifidobacterium*	4g/100g	3.4g/100g	3.5g/100g	Raw milk, inulin, food additives, gelatin, acetylated distarch phosphate, pectin, pectin gum
Water	Drinking water		—	—	—	0g/100ml	0g/100ml	0g/100ml	—

Table S2. Significantly changed KOs between days 1 and 3 in Group J

No.	KOs	Index	KOs	Index	KOs	Index	KOs
1	K02498	41	K11072	81	K10806	121	K09952
2	K03643	42	K03342	82	K13598	122	K06926
3	K03808	43	K07280	83	K06182	123	K01534
4	K07320	44	K03781	84	K06916	124	K03737
5	K00311	45	K03117	85	K09159	125	K03406
6	K01525	46	K03772	86	K05952	126	K00721
7	K02339	47	K00795	87	K05541	127	K06889
8	K09001	48	K02668	88	K02494	128	K06950
9	K09930	49	K00681	89	K00835	129	K03629
10	K11923	50	K00573	90	K00004	130	K09014
11	K04082	51	K13896	91	K03747	131	K07114
12	K07262	52	K11891	92	K03600	132	K00850
13	K02656	53	K00406	93	K13895	133	K07133
14	K03981	54	K01682	94	K13893	134	K03719
15	K04754	55	K02670	95	K02046	135	K02471
16	K05788	56	K07323	96	K09926	136	K02655
17	K03586	57	K06518	97	K06181	137	K03722
18	K03089	58	K02666	98	K07390	138	K00164
19	K05589	59	K14260	99	K07636	139	K11904
20	K06190	60	K05339	100	K07037	140	K00936
21	K04764	61	K07084	101	K12257	141	K02006
22	K10763	62	K06194	102	K07240	142	K00615
23	K07213	63	K02291	103	K02230	143	K03088
24	K06202	64	K01414	104	K10439	144	K03655
25	K07078	65	K02199	105	K13685	145	K02032
26	K07278	66	K03285	106	K07164	146	K01154
27	K03576	67	K03673	107	K01745	147	K06959
28	K03592	68	K01595	108	K08070	148	K06020
29	K07091	69	K01878	109	K01847	149	K03646
30	K09889	70	K09013	110	K05885	150	K03578
31	K08311	71	K01372	111	K07665	151	K07126
32	K06186	72	K00876	112	K03423	152	K07494
33	K03215	73	K07029	113	K07487	153	K07481
34	K03583	74	K14155	114	K01966	154	K02014
35	K03599	75	K07493	115	K01006		
36	K02686	76	K08310	116	K05995		
37	K06023	77	K07115	117	K01212		
38	K01807	78	K07102	118	K01284		
39	K05539	79	K09823	119	K01278		
40	K08304	80	K08305	120	K07003		

Table S3. Information of groups

Group	Number	Mean age (months)	Sex	
			Female	Male
W	13	57.3	9	4
J	13	54.0	7	6
T	12	56.7	4	8
Y	11	58.0	6	5

Table S4. Information of children in Biolog assays

No.	Gender	Age (months)
1	M	43
2	F	54
3	M	37
